# Reproductive steroids as potential mediators of parental–reproductive trade-offs in a brood parasitic species

**DOI:** 10.1242/jeb.250044

**Published:** 2025-05-12

**Authors:** Kathleen S. Lynch, Elisha Henson

**Affiliations:** Department of Biology, Hofstra University, Hempstead, NY 11549-1140, USA

**Keywords:** Brood parasite, Brown-headed cowbird, Estrogen, Parental care, Reproduction, Testosterone

## Abstract

Avian brood parasites display enhanced annual fecundity compared with other passerine birds. Female brood parasitic brown-headed cowbirds (*Molothrus ater*) lay a staggering estimated 40–50 eggs year^−1^. We examined how reproductive steroids mediate a possible trade-off between increased annual fecundity and parental care by comparing seasonal fluctuations in steroid profiles and follicular development in cowbirds and red-winged blackbirds (*Agelaius phoeniceus*), a related non-parasitic species. We also used gonadotropin-releasing hormone (GnRH) administration to determine whether species variation in GnRH responsivity reflects differences in behavioral phenotypes. These correlational and experimental studies are meant to test the hypothesis that reproductive steroid profiles have diverged in these two species, possibly in such a way that mediates a reproductive–parental trade-off in cowbirds. We identified several mechanisms that could enhance annual fecundity in cowbirds, and one mechanism that would do this at the cost of parental care: elevated testosterone. These results reveal that cowbirds exhibit earlier onset of breeding as measured by follicular size and estrogen concentration. Moreover, female cowbirds produce testosterone significantly quicker and more robustly in response to GnRH administration compared with red-winged blackbirds. Species divergence in seasonal steroid profiles and responsivity to GnRH, particularly with respect to testosterone, indicate the hypothalamic–pituitary–gonadal axis exhibits consequential modifications in cowbirds that may enhance reproductive output while also possibly simultaneously inhibiting caregiving behaviors.

## Teaser

**Summary:** Avian brood parasites exhibit differences compared with related, non-parasitic species in seasonal profiles of reproductive steroid hormones and responsivity of the hypothalamic–pituitary–gonadal axis. These differences may underlie the increased annual fecundity of parasitic birds.

## INTRODUCTION

More than 2300 years ago, Aristotle described what may be the first recorded observations of brood parasitic birds stealing parental care from other birds and the negative impacts this had on the host species left to care for these young (Hett, 1936). In 1859, Darwin noted that brood parasitic species were likely related to species that built their own nests, incubated their own eggs and provisioned their own young (Darwin, 1859). This was the earliest suggestion that brood parasitism is an evolutionary derived behavior that likely evolved from an ancestor with parental care (Darwin, 1859; Payne, 2005). Since these early observations, we now know that the brood parasitic strategy has arisen in roughly 1% of avian species (Davies, 2010). Although brood parasitic behavior is rare, the fact that both Aristotle and Darwin recorded their reflections about these birds highlights just how fascinating brood parasitic behavior has been to biologists and non-biologists alike, and how intriguing it is to consider ‘why’ and ‘how’ this novel behavioral phenotype evolved. This makes it even more surprising that the preponderance of studies that have investigated this behavior focused on understanding ‘why’ this behavior may have evolved, whereas there are remarkably few physiology or neurobiological studies aimed at understanding ‘how’ this behavior has evolved. Thus, brood parasitism is one example of a behavior in which biologists have yet to unite the ultimate and proximate explanations initially proposed by Tinbergen (1963). The lack of studies on the proximate mechanism underlying ‘how’ this reproductive strategy arose not only represents a gap in our knowledge, but also presents rich opportunities to study parasitic species so that we might achieve a mechanistic understanding of the stark variation in parental care that exists within and between species.

Although there is a compendium of studies from the behavioral ecology perspective, very few studies have focused on the mechanistic explanations for appearance of brood parasitic behavior. Studies that have examined mechanistic perspectives mostly focused on neuroendocrine mechanisms (Hohn, 1959; Selander, 1960; Selander and Kuich, 1963; Dufty et al., 1987; Ball, 1991; Lynch et al., 2020). Moreover, with few exceptions, most of these studies focused on parental hormones rather than reproductive hormones (Dufty and Wingfield, 1986; Saldanha and Schlinger, 1997). One study focused on reproductive steroids in male brown-headed cowbirds (*Molothrus ater*), a ubiquitous brood parasitic passerine species across North America within the Icteridae family (i.e. blackbird). This study reported distinctive hallmarks in reproductive physiology in males paired with females compared with unpaired males. Distinct differences included an extended period of elevated androgens punctuated by brief declines. Elevated androgens were parallelled by an extension in gonadal activity owing to earlier gonadal recrudescence, which resulted in the maintenance of mature gonads for significantly longer (Dufty and Wingfield, 1986). The featherless patch of skin on a female bird's abdomen called a brood patch is meant to transfer heat to developing eggs during incubation and is typically induced by hormones. But studies of estrogen and prolactin in female cowbirds report that these hormones, either individually or in combination, cannot induce brood patch formation in female cowbirds (Selander, 1960; Selander and Kuich, 1963). Yet, Hohn (1959) showed that prolactin concentration in the pituitary of breeding cowbirds is not substantially different from that of the closely related red-winged blackbird (*Agelaius phoeniceus*). It has also been demonstrated that adult cowbirds exhibit seasonal fluctuations in circulating prolactin (Dufty et al., 1987). Moreover, experimental manipulations of prolactin in cowbirds reveals an inducement of incubation-like behavior in juvenile female cowbirds that is dramatically decreased in prolactin treated adult females (Robinson and Warner, 1964). These early studies indicate that although circulating prolactin levels do exhibit seasonal fluctuations, it is likely there is less sensitivity to prolactin in cowbirds that occurs specifically during the breeding season and that this decreased sensitivity may occur in selective target tissues. Thus, early experiments suggest that selective decreases in prolactin sensitivity may be hormonally mediated (Robinson and Warner, 1964). Recent results from our lab support these early conclusions as we have found that prolactin receptor transcript abundance in cowbirds is lower in critical brain regions that regulate parental care compared with red-winged blackbirds (Lynch et al., 2020; Duque et al., 2024) and the abundance of prolactin receptor transcripts are selectively decreased via hormones, specifically mesotocin (i.e. avian homolog of oxytocin; Duque et al., 2024).

Classic parental investment theory described by Trivers (1972) predicts that maximizing reproductive output should come at a cost. In the case of the brood parasite, this cost may be a loss of parental care. The present study is rooted in this theory as we use the comparative method with the aim of identifying mechanisms that may enhance fecundity in brood parasites while concurrently inhibiting parental care. We focus our studies on brown-headed cowbirds as this species is the most well-studied parasitic blackbird. Although it is difficult to accurately measure the exact upper limit of annual fecundity of free-ranging female cowbirds, it has, nonetheless, been the subject of considerable study (Payne, 1965, 1973, 1976; Scott and Ankney, 1979, 1980, 1983; Rothstein et al., 1986; Fleischer et al., 1987; Holford and Roby, 1993). Studies report free-living female cowbirds can lay 30 eggs at a minimum (Fleischer et al., 1987; Jackson and Roby, 1992) and conservative estimates indicate older adult females lay 40–50 eggs year^−1^ (Scott and Ankney, 1980; Fleischer et al., 1987; Jackson and Roby, 1992). Still, other authors estimate free-ranging female cowbirds can exceed even this upper estimate (Jackson and Roby, 1992; Holford and Roby, 1993). In one South American cowbird species with an extended breeding season, it is estimated that free-ranging females can lay up to 120 eggs year^−1^ (Kattan, 1993, 1995). However, regardless of the exact fecundity measures, female cowbirds clearly lay at least 10-fold more eggs than other passerine birds. Thus, whereas other passerine birds optimize the number of eggs laid in a nest, cowbirds maximize the number of eggs laid.

Here, we used correlational and experimental studies to identify critical differences in reproductive steroid patterns that may have contributed to the striking divergence in annual fecundity between cowbirds and red-winged blackbirds, a species that can lay up to three broods per season, with each brood containing three or four eggs (Wright and Wright, 1944). Red-winged blackbirds were chosen for the comparisons presented here because they are closely related to the cowbird and frequently serve as a cowbird host in many parts of its range (Freeman et al., 1990). We compared seasonal steroid profiles and follicular development in these species and conducted an experimental manipulation of steroid concentrations in both species using gonadotropin-releasing hormone (GnRH) administration. This allowed us to determine whether species variation in the response to GnRH reflects differences in behavioral phenotypes of these closely related species. Overall, we hypothesize that reproductive steroid profiles have diverged in these two species, possibly in such a way that mediates a reproductive–parental trade-off in cowbirds but not in red-winged blackbirds. We predict that the seasonal timing of steroid production and robustness of GnRH response will reflect the species differences in annual fecundity.

## MATERIALS AND METHODS

### Seasonal profiles of reproductive steroids

Female brown-headed cowbirds [*Molothrus ater* (Boddaert 1783), *n*=18] and red-winged blackbirds [*Agelaius phoeniceus* (Linnaeus 1766), *n*=14] were captured using walk-in traps and mist nets in Ransom and Cass counties of North Dakota, USA, during the non-breeding and peak breeding seasons in 2021 (May–June). Traps and mist-nets were continuously monitored to ensure the birds were removed within 5 min of capture. Birds were immediately euthanized via rapid decapitation for a separate study. This allowed us to measure the diameter of the largest follicle using calipers and collect blood immediately (i.e. less than 1 min). After centrifugation, plasma was removed and stored at −80°C until assayed. Collections started 1 May (pre-breeding in North Dakota) and concluded 25 June (peak breeding). We categorized female cowbirds and red-winged blackbirds into three categories: pre-breeding, breeding onset or peak breeding. These categories were defined by observing the breeding activity (or lack thereof) of female red-winged blackbirds, the most abundant host available to cowbirds at this location. The pre-breeding condition was defined by a lack of nest building and egg laying activity present in females, whereas males were conspicuously present, singing and establishing territories (1–14 May). We captured no female red-winged blackbirds in this stage because they are either less abundant or significantly more sedentary than males at this time (Wright and Wright, 1944; Holm, 1973). The breeding onset condition was marked by the first nest building activity of a female red-winged blackbird. In this category, female red-winged blackbirds had begun building nests, but no eggs were observed in nests yet (15–22 May). The final category is the peak breeding season, which was marked by eggs observed in nests and provisioning behaviors present in female red-winged blackbirds (23 May to 25 June). These are subjective categories based on the observations of our field crew; however, these categories do coincide with the timing that female red-winged blackbirds transition from breeding to parental behaviors as described by other studies conducted at similar latitudes in North America (Wright and Wright, 1944; Holm, 1973). We measured 17-β estradiol (E_2_), testosterone (T) and follicular sizes across these breeding categories in both species.

### GnRH administration

Breeding female brown-headed cowbirds (*n*=10) and red-winged blackbirds (*n*=8) were captured using walk-in and drop-down traps in May and June 2022. Cowbirds were collected at Balcones Canyonlands National Wildlife Refuge in Marble Falls, TX, USA, at the peak of breeding season and held in captivity for 2 weeks before this study. Red-wings were collected at the peak of their breeding season at the Marine Nature Study Area, Oceanside, NY, USA, and Lido Beach Passive Nature Area, Lido Beach, NY, USA, and held in captivity for the same duration to ensure similar conditions to the cowbirds. Although the species were collected in different locations, our previous work on cowbirds has shown similar results in with birds collected at these two field sites (Duque et al., 2024; Lynch et al., 2019). Therefore, for simplicity, we collected cowbirds at the Balcones Canyonlands National Wildlife Refuge, which has pre-existing infrastructure to collect cowbirds. In addition, it has been reported that breeding stage influences the magnitude of the testosterone response to GnRH challenge in female dark-eyed junco (*Junco hyemalis*); therefore, we made sure that both species were tested at the peak of their breeding seasons (Jawor et al., 2007). Thus, cowbirds were treated with GnRH in early June and red-wings were treated with GnRH in mid-to late June. Blood samples were obtained from each female via the branchial vein within 5 min of removal from their cage. This initial blood sample was used to obtain baseline hormone measurements. Immediately following blood collection, females were injected with 1.5 µg of GnRH (Bachem catalog no. 4030773) intramuscularly in 50 μl volume dissolved in phosphate-buffered saline (Jawor et al., 2006; Burns et al., 2014; Rosvall et al., 2013, 2014). Treated birds were placed back in a cage (∼18×14×24 inches, length×width×height) after GnRH treatment so that plasma could be collected 30, 60 and 120 min after treatment. After centrifugation, plasma was removed and stored at −80°C until assayed to measure testosterone so that we could identify the immediate steroid response from the gonads, which initially produces testosterone before it is aromatized into estrogen. We then measured E_2_ with subjects that had remaining plasma: cowbirds (*n*=7), red-winged blackbirds (*n*=7).

All procedures described here were conducted in accordance with Hofstra University Institutional Animal Care and Use Committee procedures as well as federal (MB96705A-0), New York state (1181), Texas state (SPR-0521-069) and North Dakota state (GNF05428921) scientific collecting permits.

### Steroid hormone assays

Circulating T and E_2_ concentrations were quantified as described previously (Lynch et al., 2006, 2018; Pellicano, 2019; Lynch and Wilczynski, 2005, 2006, 2008). Briefly, steroids were extracted from plasma using 3 ml of diethyl ether. Extracted steroids were resuspended in assay buffer for T and E_2_ assays using ELISA kits (testosterone: catalog no. 582701, Cayman Chemical, Ann Arbor, MI, USA; 17-β estradiol: catalog no. KB30-H1, Arbor Assays, Ann Arbor, MI, USA). These kits have been validated for use in birds in previous studies (Lynch et al., 2018; Pellicano, 2019). Both steroids were measured using a single standard curve, thereby precluding an inter-assay variation measurement. In the seasonal steroid profile study, the intra-assay variation for the T assay was 15.6% and that for the E_2_ assay was 16.8%. In the GnRH study, intra-assay variation for T and E_2_ was 19.8% and 15.6%, respectively. Estradiol EIA kits have 100% cross reactivity with 17β-estradiol and all other reported cross reactivities were less than 1%. Testosterone kits have 27.4% cross reactivity with 5a-dihydrotestosterone, 18.9% cross reactivity with 5b-dihydrotestosterone, 4.7% cross reactivity with methyl testosterone, 3.7% cross reactivity with androstenedione and 2.2% cross reactivity with 11-keto testosterone. All other cross reactivities were less than 1%. The detection limit is 3.9 pg ml^−1^ for testosterone and 3.75 pg ml^−1^ for estradiol.

### Statistical analyses

In our correlative field studies, we used three separate regressions to determine how variation in the date of capture predicts largest follicle size, and concentration of 17-β estradiol and testosterone. Although these analyses reflect the correlative nature of this study, they do not allow us to draw conclusions about species comparisons. Therefore, we conducted additional analyses to directly compare variables across the species. This required a two-way ANOVA for independent samples to determine whether there was an interaction between the two species across breeding stages. This analysis was used for steroid hormone data as well as follicular size. However, we collected cowbirds in three breeding stages, whereas red-winged blackbirds could only be collected in two of these stages because they were not present at the field site yet in pre-breeding condition (or were inactive during this time); we did an additional analysis to determine whether pre-breeding hormone concentration or follicular size in cowbirds was different from the early breeding state in cowbirds and red-winged blackbirds. We used a *t*-test for independent samples and selected tests for unequal variances where data exhibited heteroscedasticity. Steroid hormone concentrations were log transformed to achieve a normal distribution. Alpha values were set at 0.05.

To identify which comparisons contributed to the significant interactions between species and timepoint, we conducted *post hoc* analyses using Bonferroni corrected paired *t*-tests with the alpha value set at 0.016. All comparisons were between the baseline timepoint (*T*_1_) and timepoints after GnRH administration (*T*_2_–*T*_4_). This *post hoc* comparison was done for each species.

We conducted additional correlational analyses using linear regression to examine the relationship between follicular size and steroid concentration in both species. These correlational analyses allow us to explore species-specific relationships between follicular growth and steroid concentration as these variables should be correlated if a typical associative breeding pattern is present.

Finally, we examined species comparisons in T and E_2_ across four timepoints in the GnRH study using 2×4 two-way ANOVA with one factor as a repeated measure. Hormone concentrations were log transformed to achieve a normal distribution. Raw data are represented in all graphs for simplicity.

## RESULTS

### Seasonal profiles of reproductive steroids

**Fig. 1.**
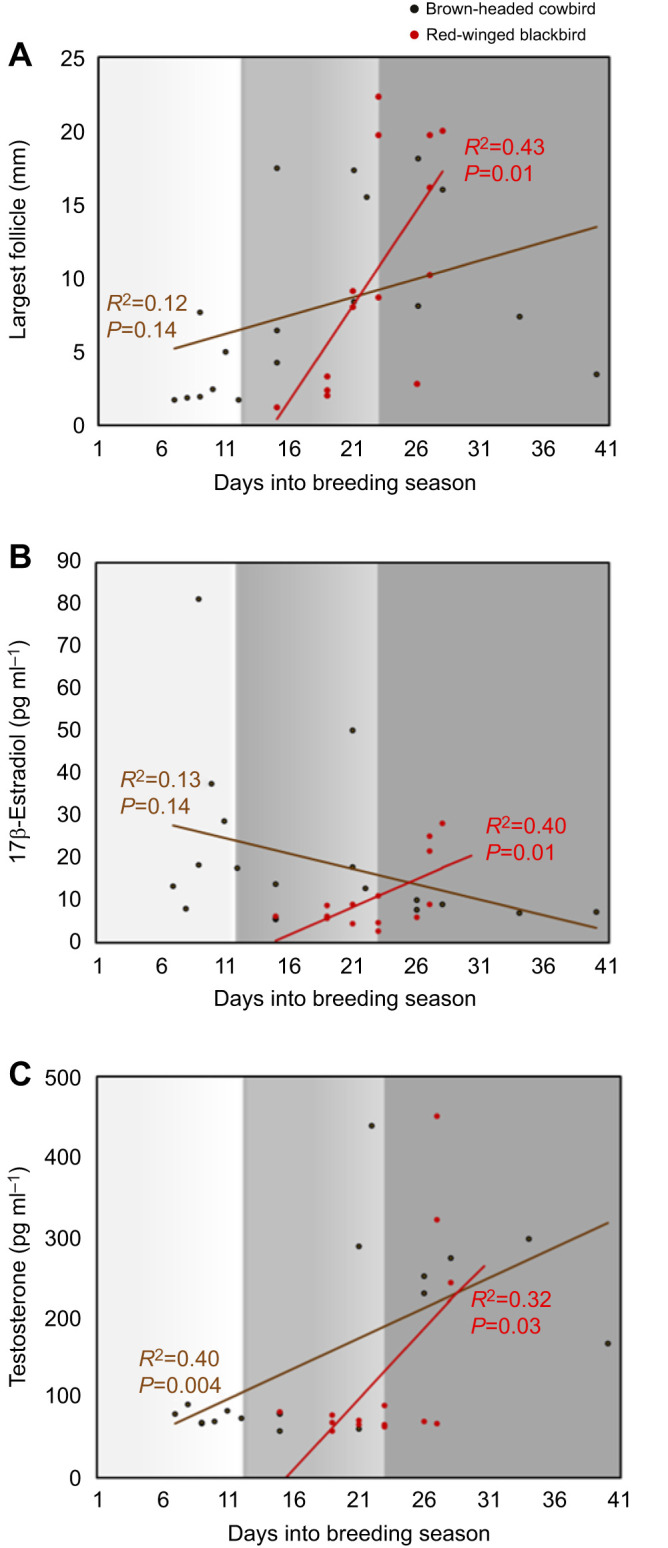
**Correlation between collection date and follicular size and hormone concentrations in female brown-headed cowbirds and red-winged blackbirds.** (A) Size of the largest follicle (mm). (B) 17-β Estradiol concentration (pg ml^−1^). (C) Testosterone concentration (pg ml^−1^). Grayscale indicates the stages of the breeding season for each of the days a sample was collected. Sample measurements were collected in three stages across 45 days: pre-breeding (light gray), breeding onset (medium gray) and peak breeding (dark gray).

We examined how variation in time within the season (i.e. pre-breeding, breeding onset and peak breeding season) predicted largest follicle size as well as circulating E_2_ and T concentrations in both cowbirds and red-winged blackbirds. In female cowbirds, there was no significant correlation between the time within the breeding season and largest follicle size (*F*_1,16_=2.9, *P*=0.14, *R*^2^=0.12; Fig. 1A), whereas there was a significant positive relationship between follicle size and time within the breeding season in female red-winged blackbirds (*F*_1,12_=9.09, *P*=0.01, *R*^2^=0.43). Likewise, variation in number of days into the breeding season that blood samples were collected did not significantly predict E_2_ concentrations in female cowbirds, but it did in female red-winged blackbirds (*F*_1,16_=2.4, *P*=0.14, *R*^2^=0.13; *F*_1,12_=8.13, *P*=0.01, *R*^2^=0.40, respectively; Fig. 1B).

In contrast, the variation in number of days into the breeding season that blood samples were collected significantly predicts circulating T concentrations in both female cowbirds and red-winged blackbirds (*F*_1,16_=10.8, *P*=0.004, *R*^2^=0.40; *F*_1,12_=5.8, *P*=0.03, *R*^2^=0.32, respectively; Fig. 1C).

With respect to the largest follicle size, results revealed a significant interaction between species and stage of breeding across onset of breeding and peak breeding stages (*F*_1,21_=6.21, *P*=0.02; Fig. 1A). Follicle size was significantly greater in red-winged blackbirds in the full breeding season compared with the early breeding season, but not in cowbirds (main effect of season: *F*_1,21_=5.18, *P*=0.03). There was no significant difference in the largest follicle size across species (main effect of species: *F*_1,21_=0.1, *P*=0.75).

With respect to circulating E_2_ concentrations, there was also a significant interaction between E_2_ concentrations between species and stage of breeding season (*F*_1,21_=3.99, *P*=0.05; Fig. 1B). However, there was no significant difference in circulating E_2_ concentrations between species or stage of breeding season (main effect of species: *F*_1,21_=0.46, *P*=0.50; main effect of season: *F*_1,21_=0.03, *P*=0.86).

With respect to circulating T concentrations, there was no significant interaction between species and stage of breeding (*F*_1,21_=1.02, *P*=0.32; Fig. 1C). There was a marginally significant difference in circulating T between species (*F*_1,21_=3.82, *P*=0.06) and a significant difference in circulating T across breeding stages (*F*_1,21_=4.84, *P*=0.04).

We collected plasma samples from cowbirds in three pre-breeding and breeding stages, whereas red-winged blackbirds could only be collected in two of these stages. Therefore, we used pairwise comparisons to analyze pre-breeding steroid hormone concentration and follicular sizes across pre-breeding and breeding onset stages between cowbirds in these stages as well as cowbirds and red-winged blackbirds. Although there was no significant difference in circulating E_2_ concentrations in cowbirds between the pre-breeding and breeding onset stages (*t*_11_=1.26, *P*=0.23; Fig. 1B), follicle size was significantly greater in cowbirds at breeding onset compared with pre-breeding stages (*t*_11_=−3.49, *P*=0.005; Fig. 1A). With respect to species comparison in these two early stages, there was a significantly higher E2 concentrations in pre-breeding cowbirds in comparison with female red-wings at the onset of breeding (*t*_11_=3.8, *P*=0.002; Fig. 1B), but no species differences in the largest follicle size at these two timepoints (*t*_11_=−0.7, *P*=0.49; Fig. 1A). With respect to circulating T concentration, there was no significant difference in T levels in the pre-breeding stage compared with the breeding onset stage in female cowbirds (*t*_11_=−1.37, *P*=0.19; Fig. 1C). There was also no significant difference in circulating T concentrations between female cowbirds in pre-breeding stages and female red-winged blackbirds at the onset of breeding season (*t*_11_=1.12, *P*=0.28; Fig. 1C).

**Fig. 2.**
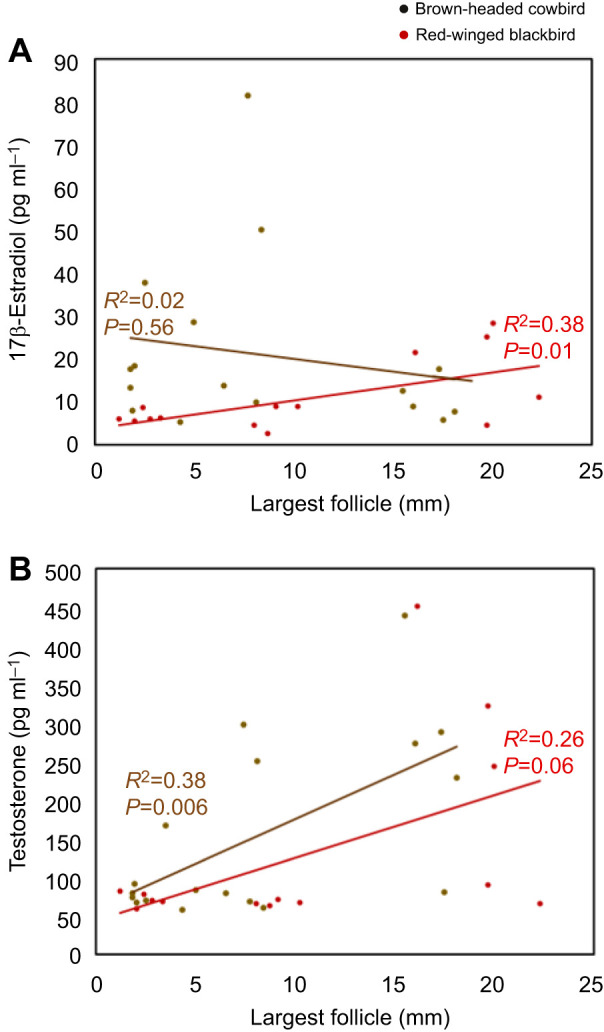
**Correlation between size of the largest follicle and circulating hormone concentrations in female brown-headed cowbirds and red-winged blackbirds.** (A) 17-β Estradiol. (B) Testosterone.

We also examined whether circulating E_2_ and T concentrations are correlated with the largest follicle size to explore whether these species have the typical pattern indicative of associative breeders in which gamete maturation and reproductive steroids are associated with one another (Nelson, 2005). There was a significant positive correlation between largest follicle size and circulating E_2_ concentrations in red-winged blackbirds but not in cowbirds (red-wings: *F*_1,12_=7.5, *P*=0.01, *R*^2^=0.38; cowbirds: *F*_1,16_=3.4, *P*=0.56, *R*^2^=0.02; Fig. 2A). In contrast, there was a significant positive correlation between largest follicle size and circulating T concentrations in cowbirds and a marginally significant correlation in red-winged blackbirds (cowbirds: *F*_1,16_=9.9, *P*=0.006, *R*^2^=0.38; red-wings: *F*_1,12_=4.2, *P*=0.06, *R*^2^=0.26; Fig. 2B).

### GnRH administration

**Fig. 3.**
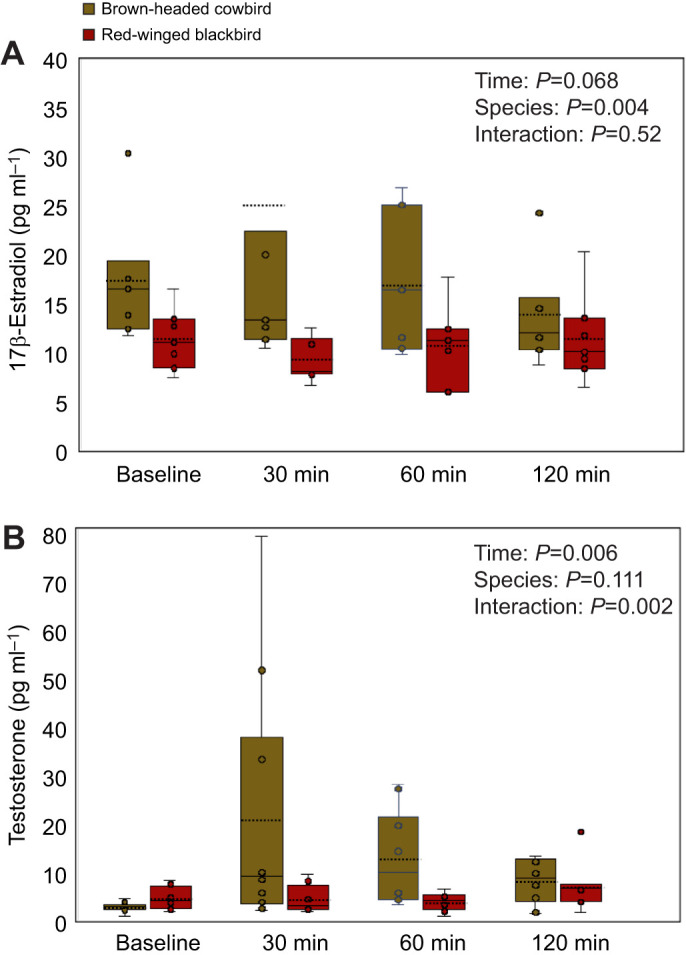
**Hormone measurements after female brown-headed cowbirds and red-winged blackbirds were treated with gonadotropin releasing hormone (GnRH).** (A) 17-β Estradiol. (B) Testosterone. Plasma samples were collected before treatment and 30, 60 and 120 min thereafter. The solid line in the box plot represents the median and dashed line represents the mean hormone concentration.

With respect to T concentrations, the results of GnRH administration revealed a significant interaction between circulating T concentration between the two species and across the four timepoints (interaction between species and timepoints: *F*_3,48_=5.3, *P*=0.002; Fig. 3B). There was a significant main effect of the timepoint at which blood was sampled as there was a significant difference in circulating T concentrations across the four timepoints after GnRH treatment (*F*_3,48_=4.7, *P*=0.006). However, there was not a significant main effect of T differences across the two species (*F*_1,16_=2.8, *P*=0.11).

To identify which comparisons contributed to the significant interactions between species and timepoint, we conducted *post hoc* analyses using Bonferroni corrected paired *t*-tests with the alpha value set at 0.016. All comparisons were between the baseline timepoint (*T*_1_) and timepoints after GnRH administration (*T*_2_–*T*_4_). With respect to circulating T in female cowbirds, there was a significant difference at the 0.016 level for all timepoints after GnRH administration (*T*_2_–*T*_4_) when compared with the *T*_1_ baseline (*T*_1_–*T*_2_: *t*=3.07, *P*=0.013; *T*_1_–*T*_3_: *t*=4.9, *P*=0.0008; *T*_1_–*T*_4_: *t*=4.3, *P*=0.0002). On the contrary, with respect to circulating T in female red-winged blackbirds, there were no significant differences at any of the timepoints compared with the *T*_1_ baseline (*T*_1_–*T*_2_: *t*=0.41, *P*=0.68; *T*_1_–*T*_3_: *t*=0.96, *P*=0.36; *T*_1_–*T*_4_: *t*=1.7, *P*=0.13).

With respect to E_2_, there was no significant interaction between circulating E_2_ concentration between the two species and across the four timepoints (*F*_3,36_=0.75, *P*=0.52; Fig. 3A). There was no main effect of the timepoint at which samples were collected as there was no significant difference in circulating E_2_ concentrations across time after GnRH treatment (*F*_3,36_=0.5, *P*=0.68). In contrast, there was a significant main effect of species as there were significantly greater E_2_ concentrations in brown-headed cowbird females compared with female red-winged blackbirds (*F*_1,12_=11.8, *P*=0.004).

## DISCUSSION

Reproductive steroids, particularly testosterone, have significant fitness consequences and are under selection (McGlothlin et al., 2010; Burns et al., 2014), which makes them excellent candidates to serve as systems that drive phenotypic evolution. Moreover, because reproductive steroids are targets of selection, they may drive trade-offs that ultimately shape life history strategies (McGlothlin et al., 2010), which could potentially contribute to the evolution of novel behavioral phenotypes such as brood parasitism.

In 1929, Friedmann suggested that cowbirds are in an evolutionary transition from determinate to indeterminate egg layers (Friedmann, 1929), which would maximize reproductive output and have a clear adaptive significance for the brood parasitic lifestyle. However, even with indeterminate egg laying, free-ranging cowbirds still lay eggs in clutches (Payne, 1965; Scott and Ankney, 1983). The ovaries and oviducts of female cowbirds do not regress between clutches, which limits the average period between clutches to only 2 days, with some females missing only a day between clutches (Scott and Ankney, 1983). These physiological adaptations allow cowbirds to lay up to 30–50 eggs year^−1^ and possibly more (Scott and Ankney, 1980; Fleischer et al., 1987; Jackson and Roby, 1992). Here, we compared steroid hormone profiles in a parasitic and non-parasitic blackbird to determine whether these profiles reflect species differences in annual fecundity and whether profiles of reproductive steroids could possibly mediate a trade-off between increased reproductive output and a loss of parental care in the brood parasitic cowbird.

One mechanism we reveal here that likely contributes to the enhanced reproductive output in female cowbirds is the earlier initiation of follicular development in female cowbirds compared with female red-winged blackbirds (Fig. 1A). Female cowbirds have large follicles in the pre-breeding season, with one female reaching 7.7 mm before breeding has even begun for female red-winged blackbirds (Fig. 1A). Moreover, at the onset of the breeding season, female cowbirds still maintain larger follicles compared with red-winged females (Fig. 1A), but this species difference reverses during the peak breeding season, when laying whole clutches is ongoing for the female red-winged blackbird (Fig. 1A). This pattern in follicular development suggests that female cowbirds can jumpstart their reproductive activity so that they may be ready to parasitize red-wing nests as soon as they become available. However, female red-wings are not the only nesting passerine species available at this location, so it is possible that female cowbirds initiate their reproductive physiology to coincide with some other host not examined in this study. But the other common passerine species nesting in the location include yellow-headed blackbirds (*Xanthocephalus xanthocephalus*), common grackles (*Quiscalus quiscula*) and marsh wrens (*Cistothorus palustris*), all of which are rarely or never parasitized by cowbirds (Picman, 1986; Ortega and Cruz, 1988, 1991; Dufty, 2000). In addition, cowbirds can travel long distances to parasitize nests (Rothstein et al., 1984; Thompson, 1994) and therefore their reproductive physiology may initiate to coincide with a host species nesting far outside the marsh. However, red-winged blackbirds are still the most abundant host species available within this region (Besser and Brady, 1984) and are among the species cowbirds prefer to parasitize (Freeman et al., 1990; Clotfelter and Yasukawa, 1999). Thus, there is an abundance of female red-winged blackbirds at this study site and because cowbirds prefer them as hosts for their young (Woolfenden et al., 2003), cowbirds should initiate breeding when red-wings make nests available. However, these data suggest that female cowbirds initiate follicular development before those nests are available. Consequently, female cowbirds are reproductively active before their main hosts have even completed nest building and this jumpstart likely improves the cowbirds annual reproductive output.

The conclusion that female cowbirds jumpstart the breeding season is further supported by the pattern of circulating E_2_. Female cowbirds exhibit higher E_2_ concentrations during breeding onset compared with female red-winged blackbirds. But, just as was the case in our follicular maturation data, this pattern reverses once female red-wings enter into peak breeding patterns. Interestingly, however, both female cowbirds and red-wings exhibit similar patterns of T production throughout the breeding season. However, the pattern of T and E_2_ production across the breeding stages are similar in the female red-wings but not in female cowbirds. Circulating T and E_2_ exhibit interacting trends in the female cowbird, suggesting that there may be species differences across the breeding stages with respect to the aromatization of T into E_2_.

There is also a species difference between cowbirds and red-winged blackbirds in the association between estrogen and follicular development. This is remarkable for two reasons. First, most seasonally breeding songbirds (with few exceptions) are textbook examples of associated breeders in which gamete development coincides with reproductive behaviors and the steroids that regulate reproduction (Nelson, 2005). However, in this study, only female red-wings displayed an association between follicular development and estrogen. This verifies our previous study that reported a similar disassociation between estrogen and follicle size in female cowbirds (Lynch et al., 2018). Together, these studies suggest that the reproductive physiology of female cowbirds has shifted away from the standard associated breeding pattern that is typical of most other breeding songbirds. This shift may be associated with the cowbirds transition to indeterminate egg laying, which promoted increased reproductive output. Second, the other significance of this result can be seen when considering the seasonal estrogen profile alongside the seasonal pattern of testosterone in these two species. We also uncovered a species difference in the pattern of estrogen and testosterone across both follicular development and the breeding stages. There was a positive correlation between testosterone and follicular maturation in both species as they progressed across the breeding stages (Fig. 2B). However, female cowbirds displayed a significant correlation between testosterone concentration and follicular maturation whereas female red-winged blackbirds exhibited a marginally significant correlation between these measures of reproductive physiology (*P*=0.06). Moreover, in female cowbirds, but not female red-wings, T exhibited a complete reversed pattern compared with the seasonal estrogen profile (Fig. 2). Thus, the two species exhibit opposing patterns with respect to how testosterone and estrogen fluctuations occur across the season and follicular development. Because testosterone is aromatized into estrogen, the opposing estrogen profiles in these species suggest there may be species differences in how or when this happens. Although it is speculative, it is possible that cowbirds aromatize testosterone on demand as opportunities to parasitize nests arise. Such a pattern in aromatase activity would make it hard to detect an association between estrogen and follicular development while making an association between testosterone and follicular development apparent. More importantly, however, it would lead to a pattern of heightened testosterone in female cowbirds relative to females of other species, which is the pattern we see in both the seasonal profiles and the experimental manipulation studies presented here. In addition, this elevated testosterone in the female cowbirds would likely remain elevated for a prolonged timeframe to allow cowbirds to continue to lay eggs throughout the breeding season. This elevated testosterone would promote robust reproductive activity but may also inhibit parental behavior (Clotfelter et al., 2004; Ketterson et al., 2005; Rosvall, 2013).

Although it is peculiar that female cowbirds initiate breeding physiology before their preferred hosts have started breeding and have a breeding pattern indicative of a non-associative breeder, it is possible that these patterns are suggestive of socially regulated reproductive physiology. For instance, the follicle development and elevated estrogen occurring in the pre-breeding stage of female cowbirds is also occurring at a time when male red-winged blackbirds are abundant and are actively engaging in territorial song (Wright and Wright, 1944). Previous experiments showed increasing circulating estrogen in female cowbirds exposed to songs of either male red-winged blackbird or male cowbirds (Lynch et al., 2018). Thus, exposure to songs of red-winged blackbirds can promote hypothalamic–pituitary–gonadal (HPG) activity in female cowbirds possibly because cowbirds are well-known eavesdroppers that attend to the social cues of potential host species as a means of locating nests (Wiley, 1988; Clotfelter, 1998; Monk and Brush, 2007; Janecka and Brush, 2014). This is referred to as the host activity hypothesis (Banks and Martin, 2001; Robinson and Robinson, 2001). Social regulation of the HPG axis was initially identified by Lehrman and colleagues nearly 60 years ago (Lehrman, 1965) and has since been reported to occur in both sexes, across vertebrate species, and within a variety of social behaviors (see Watts, 2020 for review), including in male and female cowbirds (Dufty and Wingfield, 1986; Lynch et al., 2018). It is possible that cowbirds are especially sensitive to social regulation of the HPG axis as the social cues of countless heterospecific species should be biologically relevant for the cowbird, as predicted by the host activity hypothesis. The extent of heterospecific social cues and the sensitivity of the cowbirds' HPG axis after exposure to these social cues remain unknown. However, clearly heterospecific song, particularly songs of the male red-winged blackbird, promote HPG activity in female cowbirds (Lynch et al., 2018), and this social regulation of reproductive physiology may serve as a mechanism that allows female cowbirds to orchestrate their reproductive physiology to match the breeding activity of their hosts.

The possibility that cowbirds are especially sensitive to social regulation of the HPG axis, as predicted by the host activity hypothesis, is supported by the results of the GnRH challenge in which we show species differences in the timing of HPG responsivity to this secretagogue. These results reveal that female cowbirds respond faster and more robustly after GnRH administration as compared with female red-winged blackbirds (Fig. 3A). It takes roughly 30 min for female cowbirds to exhibit increased circulating testosterone whereas female red-wings require over an hour for a marginal increase to occur. Moreover, there is a species difference in estrogen concentrations in which female cowbirds have greater circulating estrogen concentrations compared with female red-wings, even at the baseline timepoint before GnRH was administered. The results of these experimental manipulations suggest that female cowbirds have heightened sensitivity to GnRH, which allows them to respond quickly to this secretagogue. This would allow cowbirds to quickly initiate a physiological response when breeding opportunities arise. Again, although it is speculative, this pattern supports the premise that the reproductive physiology needed to enhance fecundity may occur on-demand in female cowbirds, as heightened responsivity to secretagogues could allow cowbirds to match their reproductive physiology to social cues associated with nesting opportunities.

Overall, both the correlational and experimental studies presented here suggest that how and when testosterone is aromatized into estrogen and how testosterone responds to GnRH is different between cowbirds and non-parasitic blackbirds. These differences point to testosterone as a possible mediator that enhances reproductive output at the expense of parental physiology and parental care. The role of testosterone in facilitating reproductive behavior while also inhibiting parental behaviors is well established in both male and female birds (Clotfelter et al., 2004; Ketterson et al., 2005; Rosvall, 2013; for review, see Lynn, 2016). In male birds, testosterone promotes courtship and aggressive behaviors involved in sexual selection while also reducing paternal behavior (Ketterson and Nolan, 1999; Wingfield et al., 2001; McGlothlin et al., 2007; O'Neal et al., 2008). In some species, males exhibit a pattern in which testosterone levels decline at the onset of the parental phase when nesting-related stimuli act to promote an increase in hormones, such as prolactin, that regulate caregiving behaviors (Buntin et al., 1996; for review, see Lynn, 2016). Moreover, in some males, administration of testosterone during the parental stage results in reduced incubation and nest abandonment (Oring et al., 1989). In contrast, exactly how testosterone mediates switching between reproduction and parenting is more variable in females. It is clear, however, that female birds investing more into reproduction often exhibit an associated decrease in maternal behavior, and in some cases, testosterone can mediate this trade-off (Ketterson et al., 2005; O'Neal et al., 2008; Cain and Ketterson, 2012; Rosvall, 2013). For example, testosterone treatment in some species of female songbirds increases reproductive and aggressive behaviors, but often at the expense of certain measures of maternal behaviors, including delayed egg laying, reduced incubation or provisioning, reduced brooding and nest defense, and even reduced hatching success (Clotfelter et al., 2004; Rutkowska et al., 2005; O'Neal et al., 2008; Veiga and Polo, 2008; Gerlach and Ketterson, 2013; Rosvall, 2013). Moreover, female dark-eyed juncos treated with GnRH in the pre-breeding season will exhibit a robust testosterone response but not if they are treated with GnRH when they are feeding nestlings (Jawor et al., 2007). Rosvall (2013) concluded that the varied effects of reproductive steroids on female parental care are likely related to specific life history of the species and the relative importance of competition versus maternal care for those females. This conclusion can be appropriately applied to female cowbirds because this species does not ever feed nestlings or provide any form of caregiving to their young, and consequently, there should be no period throughout the breeding season in which they are resistant to elevated testosterone. In fact, maintaining consistently elevated testosterone would be a mechanism that allows them to lay eggs indeterminately, thereby increasing annual fecundity, but doing so would come at the expense of parental behavior.

Although these studies indicate there are mechanistic shifts in HPG activity of female cowbirds that would facilitate increased fecundity, we cannot presume whether this shift is a cause or consequence of a loss in parental care. We do, however, consider these results alongside previous comparative studies of four transcripts involved in regulating parental care – prolactin receptor, arginine vasotocin (avian homolog of vasopressin), mesotocin (avian homology of oxytocin) and galanin – between the same two species studied here (Duque et al., 2024). It was reported that these four transcripts exhibit lower abundance in female cowbirds in at least one critical brain region involved in regulating parental care and for some of these transcripts, lower abundance occurred in all brain regions examined (Lynch et al., 2020; Duque et al., 2024). Most importantly, however, mesotocin (i.e. oxytocin) decreases prolactin receptor transcript abundance in cowbirds but not red-winged blackbirds, indicating that hormones reduce receptors involved in parental physiology in a species-dependent manner (Duque et al., 2024). Consequently, future studies need to focus on whether testosterone is also able to modify prolactin receptors and other critical parental care-related receptors in the cowbird brain. A study reported that short-term testosterone treatment does not alter prolactin receptors in treated and untreated dark-eyed junco females (Schoech et al., 1998). However, these modifications might occur over short (i.e. seasonal) or long (i.e. evolutionary) timescales or may be species-specific, as our previous study revealed (Duque et al., 2024). Thus, studies that seek to understand whether the chicken (i.e. reproductive maximization) or the egg (i.e. loss of parental care) came first are still needed.
